# The Co-Occurrence of Species and the Co-Diversity of Sites in Neutral Models of Biodiversity

**DOI:** 10.1371/journal.pone.0079918

**Published:** 2013-11-11

**Authors:** Paulina Trejo-Barocio, Héctor T. Arita

**Affiliations:** 1 Posgrado en Ciencias Biológicas, Universidad Nacional Autónoma de México, México, D. F., México; 2 Centro de Investigaciones en Ecosistemas, Universidad Nacional Autónoma de México, Morelia, Michoacán, México; Centre National de la Recherche Scientifique, France

## Abstract

Patterns of co-occurrence of species are widely used to assess the fit of ecological neutral models to empirical patterns. The mathematically equivalent patterns of co-diversity of sites, in contrast, have been considered only indirectly and analyses normally are focused on the spatial distribution of species richness, rather than on the patterns of species sharing. Here we use two analytical tools (range-diversity plots and rank plots) to assess the predictions of simple neutral models in relation to patterns of co-occurrence and co-diversity. Whereas a fully stochastic null model predicts zero average among species and among sites, neutral models generate systems with low levels of covariance among species and high levels of positive covariance among sites. These patterns vary with different combinations of dispersal and speciation rates, but are always linked to the shape, symmetry, and spread of the range-size and species-richness frequency distributions. Non-homogeneous patterns of diversity and distribution arise in neutral models because of the spatial arrangement of sites and their concomitant similarity, which is reflected also in the spread of the range-size frequency distribution. The nearly null covariance among species, in contrast, implies low variance in species richness of sites and very slim frequency distributions. In real world assemblages of Mexican volant and non-volant mammals, patterns of range-size and species-richness frequency distribution are similar to those generated by neutral models. However, when the comparison includes the covariance both for species (co-occurrence) and for sites (co-diversity), empirical patterns differ significantly from the predictions of neutral models. Because of the mathematical links between the covariance in the distribution of species and the variance of species-richness values and between the covariance in species sharing among sites and the variance of range-size values, a full understanding of patterns of diversity calls for the simultaneous analysis of co-occurrence and co-diversity.

## Introduction

Heterogeneity in the distribution of species and their diversity is one of the most obvious patterns in macroecology. Because the size and location of geographical ranges varies much among species, different taxa are found in different places, and patterns of species richness arise as a consequence. By studying spatial and temporal patterns in the distribution of species, biologists try to understand the mechanisms that generate and maintain biological diversity at different spatial and temporal scales [1]. Intuitively, intrinsic differences among species should contribute to higher levels of diversity. If species have different demographic traits, dispersal capabilities, and habitat requirements, those differences inevitably lead to variation in the size and structure of their ranges, and to a concomitant heterogeneity in the distribution of diversity. An important body of research in ecology is aimed at examining differences among species to understand patterns of co-occurrence, and thus, patterns in the distribution of diversity [2], [3].

Ecological and evolutionary models have been proposed to test hypotheses regarding the distribution and diversity of species. Null models were conceived to simulate assemblages in which species distribute randomly, but retaining some of the basic differences among them (*e.g*., the size of their ranges). The objective of null models is to test for ecological processes, such as species interactions, by generating statistical distributions in the absence of the process of interest and by comparing empirical patterns to that distribution [4]. Other models have examined the dynamics of diversification through stochastic birth and death processes in which no difference is considered among species [5]. In yet another type of stochastic models, researchers conceived processes based on the non-intuitive assumption of no differences in biological traits among individuals of different species [6], [7], [8]. These models were based on Kimura's neutral models of genetic evolution [9], so they became to be known as ecological neutral models. Neutral models are based on stochastic processes that simulate the origin, dispersal, and death of individuals of different species, with all individuals having exactly the same biological traits regardless of their species. Species assemblages produced by neutral models, in its original form or in modern, more sophisticated versions, are surprisingly similar to real life ecological communities at different spatial scales [8], [10], [11], [12], [13], a fact that prompted the proposition of Hubbell's Neutral Theory of Biodiversity and Biogeography to explain real life patterns of diversity and distribution as the result of purely stochastic processes [8]. Today, after a little more than a decade since the publication of Hubbell's monograph, the relative role of stochastic processes in shaping local communities and regional assemblages of species is hotly debated [14], [15], [16] and the power of neutral models in explaining real world patterns is still the subject of intensive scrutiny [17], [18], [19], [20].

Comparisons of predictions of neutral models with real life assemblages of species have been based mostly on the frequency distribution of abundance among species, the species-area relationship, the spatial distribution of species richness, and the spatial turnover of species [13], [14], [15], [21], [22]. Less frequently, patterns based on the occurrence and co-occurrence of species have been also used to test predictions of neutral models, particularly at large, macroecological, scales [11], [23], [24]. Occurrence is normally examined through the analysis of the range-size frequency distribution (RSFD), and co-occurrence can be quantified with various indices that measure the degree of association or segregation in the spatial distribution of species [2], [3], [24]. A more direct measure of co-occurrence is the covariance between pairs of species in terms of the number of sites in which the two ranges overlap [23], [25], [26]. A positive covariance indicates association between the two species, whereas a negative covariance suggests spatial segregation. The overall pattern of co-occurrence can be quantified by averaging all pairwise covariance values across all species in the assemblage [25], [26], providing additional parameters to contrast predictions of neutral models with real-life data.

If patterns of covariance quantify the co-occurrence of species, then, in exactly the same way, covariance among pairs of sites in terms of the number of species that they share can be used to measure what has been called the co-diversity of sites [23]. Just as the co-occurrence of species is determined by patterns of association or segregation, the co-diversity of sites depends on the degree of similarity or differentiation among localities in terms of species composition [27]. A positive covariance between two sites indicates that they share more species than expected by chance, whereas a negative covariance is evidence of a differentiation in species composition higher than expected by chance. As with species, covariance by sites can be averaged across all sites to produce an overall measure of co-diversity. In contrast with the co-occurrence of species, the co-diversity of sites has not been widely adopted as a comparing parameter for null and neutral models [23]. Instead, diversity is routinely examined with the spatial pattern of species richness, but parameters such as the species richness frequency distribution (SRFD) and the co-diversity of sites have been basically ignored as testing variables.

It could be argued that the analysis of patterns of covariance among species (co-occurrence) and among sites (co-diversity) should be central in testing predictions of neutral models. By the very definition of neutral models, the distribution of species is generated through random processes, so ranges should be independent of each other. Consequently, the expected average covariance between species should be zero, providing a specific prediction to be tested. In contrast, the expected average covariance and the frequency distribution of pair-wise covariances between sites cannot be easily derived from the assumptions of spatially-explicit neutral models. This is because the probability of species migrating to localities differs among sites as a function of distance, so sites are not independent and the average covariance between them is bound to be different from zero. Thus, by using the patterns of covariance both for species and for sites, modelers can generate a complete battery of tests to contrast the results of the models against real world patterns.

In this paper, we examine the patterns of co-occurrence and co-diversity under different conditions for simple neutral models. We also use examples of continental assemblages of mammal species to compare the predictions of the models with real world patterns. We examine the output of the neutral models and the empirical data to illustrate the use of range-diversity plots [25], [26] and rank plots as powerful visual and analytical tools in quantifying patterns of diversity and distribution. Our expectation was that a totally randomized model would generate patterns with null average covariance both for species and for sites, but we expected that neutral models could generate non-zero covariances for sites, depending on the parameters of dispersal of the models, but not for species.

## Methods

### Neutral model

We implemented a model with neutral community dynamics based on Hubbell's neutral theory of biodiversity [7], [8]. Our model consisted of a spatially explicit metacommunity, established on a homogeneous space, and formed by a set of species of the same trophic level having exactly equal demographic traits and showing no biotic interactions [11], [28]. These conditions were attained by allowing each individual in the metacommunity to be born, die, migrate, and speciate with exactly the same probability as any other individual, regardless of species identity. The modeled space was a square grid of 256 cells, each one harboring at least one individual at all times.

At the start of each simulation, 8,500 individuals were randomly located on this grid and assigned at random to one of 143 species. These initial conditions also represent a reference point corresponding to a system in which the distribution of species and the species richness of sites are totally randomized. These conditions are biologically unreasonable but serve as an extreme null model in which the expected covariances, both for species and for sites, should be null. Thus, the examination of these initial conditions represents a point of comparison for interpreting the conditions at the end of the simulations.

As dictated by the neutral theory, dynamics of the system followed very simple rules: In each simulation cycle, every individual could die with a fixed probability *d* (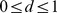). Dead individuals were replaced instantaneously with another individual of the same or of a different species, every species having the same probability of being chosen, so *d* is best described as a death-birth parameter. This death-birth process maintained the number of individuals constant through the simulations, thus complying with the zero-sum assumption of classic neutral models [7], [8]. A dispersal parameter *m* (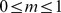) defined the probability of an individual moving from its original cell to another one at each time step, with higher probability for sites closer to the source cell following a linear function with distance. Finally, a parameter of point mutation speciation 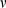 (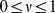) [8], defined the probability that an individual changed its species membership (thus “speciating”) during one time step. Each simulation consisted of 2,000 cycles of these processes. For the different simulations, we varied the speciation probability (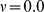, 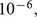 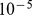, 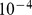, 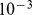 per unit time) and the dispersal parameter (*m* = 0.0. 0.25, 0.5 per unit time), while keeping the death-birth probability constant (*d* = 0.01 per unit time).

### Empirical cases

We examined patterns of co-distribution and co-diversity for two assemblages of mammalian species at different scales to contrast results of the neutral model with real-world cases. The first assemblage was a set of 136 species of Mexican bats distributed over a grid of 824 0.5×0.5 degree quadrats (approximately 2840 km^2^ at the latitude of Mexico). The second set included the non-volant mammals of a grid of 62 0.5×0.5 degree quadrats located in Central Mexico, in the limit of the nearctic and neotropical biogeographic regions. In both cases, information on the distribution of species was gathered from museum specimens and data from the primary literature [29], [30].

### Presence-absence matrices and range-diversity plots

Output from each simulation and from the empirical cases was transferred to species × sites presence-absence matrices. The distribution of *S* species in *N* sites can be summarized in an 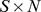 matrix with elements 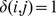 if species *i* is present in site *j*, and 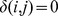 otherwise. We used the R script in reference [26] to extract information on the distribution of species and the diversity of sites through row and column sums of the matrix, respectively. The range size of each species(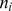), defined here as the number of cells in which that species occurs, equals the sum of elements of the matrix along the row corresponding to that species (R-analysis). In the same manner, the species richness of a given site (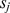) can be calculated as the sum of elements of the matrix along the column corresponding to that site (Q-analysis). By combining R- and Q-analyses, additional parameters can be computed: the range-richness of a species (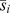) is the average number of species occurring in the sites where species *i* is present and the per-site range size (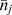) is the average number of sites occupied by the species occurring in site *j* [25], [26].

By plotting 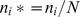 (the range size of species proportional to the total number of sites) vs 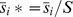 (the proportional species richness of sites in which species *i* occurs), distribution and diversity can be visualized in a single plot in which each point represents a species. Equivalently, with a plot of 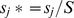 (proportional species richness) vs 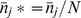 (proportional per-site range size) parameters of diversity and distribution can be displayed simultaneously by sites. These two types of range-diversity plots (RD plots) are necessary to have a complete depiction of a system, as they summarize the complementary but independent patterns of diversity and distribution by species and by sites [25], [26].

Range-diversity plots are particularly suited for analyses of co-occurrence and co-diversity because of the statistical relationship between covariance and the parameters of diversity and distribution used in the plots. In an analysis by species, it can be shown that 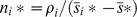, where 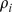 is the average covariance of species *i* with all species and 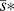 is the average proportional species diversity in all sites. In a RD plot depicting 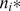 as a function of 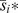, species with the same average covariance arrange along hyperbolic curves following this equation, and species with average covariance equal to zero arrange along a vertical line coinciding in the x-axis with the average proportional species richness of all sites [25], [26]. Points to the right of this vertical line correspond to species with positive average covariance (overall positive association), and points to the left show species with negative average covariance (overall segregation).

In exactly the same mathematical way, range-diversity plots by sites show the average covariance through the relationship 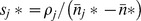. In this case, 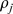 is the average covariance of site *j* with all sites, and 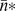 is the average proportional range size of all species. In the plot by sites, points to the left of a vertical line coinciding with 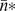 correspond to sites with negative average covariance (differentiation), and points to the right of the line show sites with average positive covariance (similarity). An important property of presence-absence data is that for any system the average proportional range size of all species equals the average proportional species richness of all sites (that is, 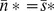). Moreover, these proportional averages are also equal to the proportional fill of the matrix, that is, the number of occurrences (ones) in the matrix in proportion to the size of the matrix, 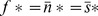 = 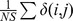, where the sum is across the whole matrix. As a consequence of this equality, the position of the vertical line of zero covariance is the same in RD plots both for species and for sites, coinciding in the x-axis with the value of 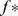 [25], [26].

### Rank distribution and rank diversity plots

**Figure 1:**
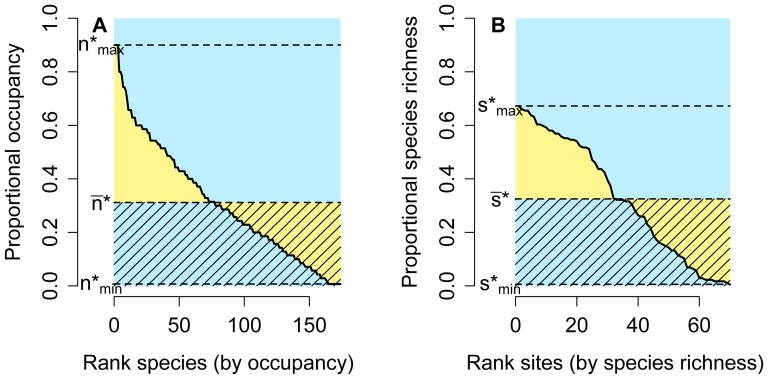
Rank distribution and rank diversity plots. Hypothetical example showing species ranked by their occupancy or range size (A), and sites ranked by their species richness (B). The area of the blue rectangle equals the number of species (in A) or the total number of sites (in B). Yellow areas measure the unevenness of the range-size (A) or species-richness frequency distributions (B), and the shaded areas are equal to the average species richness of sites (in the plot by species, A) or the average range size of species (in the plot by sites, B).

The range-size frequency distribution (RSFD) is generally depicted using histograms [31], [32]. Here we show that the alternative rank distribution plots, sequences of species ordered from the most widespread to the most restricted and showing their proportional range size (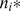), display more visual and quantitative information amenable to comparisons of co-occurrence patterns (Fig. 1a). First, the area under the curve equals the summation of all proportional ranges, but that sum also equals the average species richness among sites [25]. Second, the area under the line defined by the average proportional range (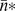) also equals that summation (because it is the average multiplied by the number of species) and also show the average richness of sites. Third, the area of the whole plot is equal to 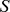, the total number of species; this can be seen by noticing that the boundaries for the plot are 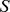 in the abscissas and 1 in the ordinates, so the area is clearly equal to 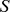. Another way of seeing this is by imaging a system in which all species occur in all sites; in that case, the rank curve would be a horizontal line along the top of the plot, so 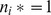 for all species, and the area under the curve would be 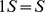. Finally, the area between the horizontal line marked by 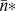 and the curve is a measure of unevenness of the RSFD; a system with maximum evenness would have all species occupying exactly 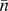 sites and would generate rank plots with no yellow area. Notice that, because the areas of the blue rectangle equals 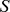, dividing the yellow area by the blue area is equivalent to computing the average absolute deviation of range size values from the average, so the parameter 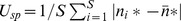 is a measure of unevenness in the range size frequency distribution, and rank distribution plots provide a visual representation of such variable and of the variance in range size.

With exactly the same reasoning, rank diversity plots can be drawn by depicting the species richness frequency distribution (SRFD) as a sequence of sites arranged by their species richness (Fig. 1b). Because 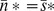 [25], the central location of the curves has to be the same in corresponding rank distribution and rank diversity plots, but the unevenness can vary independently for sites and species. In this case, unevenness is measured as 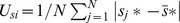, and, again, the yellow area in Fig. 1b, relative to the blue area, is a visual representation of this variable and of the variance in species richness.

### Single-parameter measures of co-distribution and co-diversity

We used Schluter's variance ratio test, 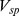 [33] to quantity in a single parameter the complex patterns of co-occurrence of species. The test is based on the fact that the variance in species richness among sites equals the sum of the variance/covariance matrix of species, so 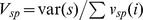, where 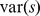 is the variance in species richness among sites and 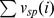 is the summation of the variance within the ranges of species, is a measure of covariance among species [33]. If the average covariance among species equals the average variance within ranges, then 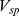 = 1; 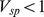 indicates negative average covariance, and 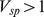 means that the average covariance is positive. We also used the mathematically equivalent parameter 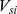 (a ratio of the variance in range size and the sum of variances in species richness of sites) to measure the significance of the covariance among sites [23], [25].

We used Pearson's 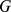 as a measure of skewness or asymmetry in the frequency distribution of range size and species richness. 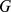 equals 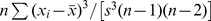, where the 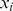 are the observations of range size (for species) or species richness (for sites), 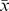 and 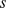 are the mean and the standard deviation of these observations, and 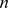 is the sample size, that is the number of species or the number of sites. Positive values of 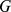 indicate a concentration of values in the left part of a histogram and a long tail to the right; negative 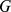 values indicate left-skewed curves.

## Results

### Initial conditions

**Figure 2:**
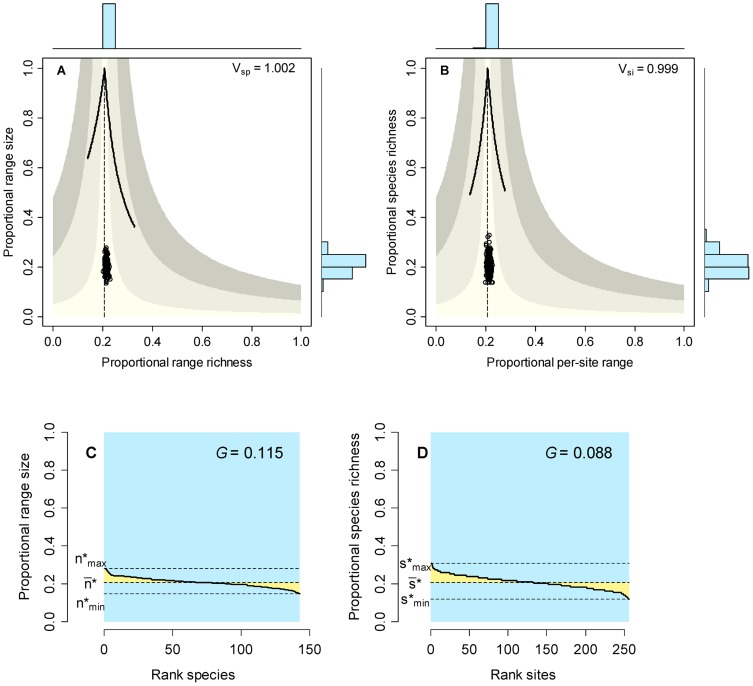
Initial conditions of the neutral models described in the text. Range diversity plots by species (A) and by sites (B) show the values of Schluter's variance ratio index. Vertical dashed lines correspond to the value of 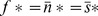; solid curves show maximum possible values in richness and range size, and shaded areas show the average covariance of species and of sites (from the vertical line outward, limits are covariance +/−0.01, 0.05, 0.1. Rank plots depict the range-size (C) and species richness (B) frequency distributions and the value of Pearson's measure of skewness; colors as in Figure 1.

The random allocation of 8,500 individuals to 143 species and 256 sites generated a presence-absence matrix with low fill (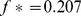) which implied that mean range size was 20.7% of the cells (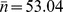) and average species richness was 20.7% of species (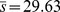). Variation around the mean was very low, generating narrow and symmetrical range-size and species-richness frequency distributions (right-hand histograms in Fig. 2A and B, and rank plots in Fig. 2C and D). Species showed no association or segregation (mean covariance  = 0.0011, Schluter's 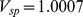), and sites showed no similarity or differentiation (mean covariance <0.001, 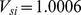). These patterns produced RD plots in which points arranged along the vertical central lines (zero mean covariance), well inside the area of mean covariance <+/−0.01 (Fig. 2A and B).

### Neutral models

**Figure 3:**
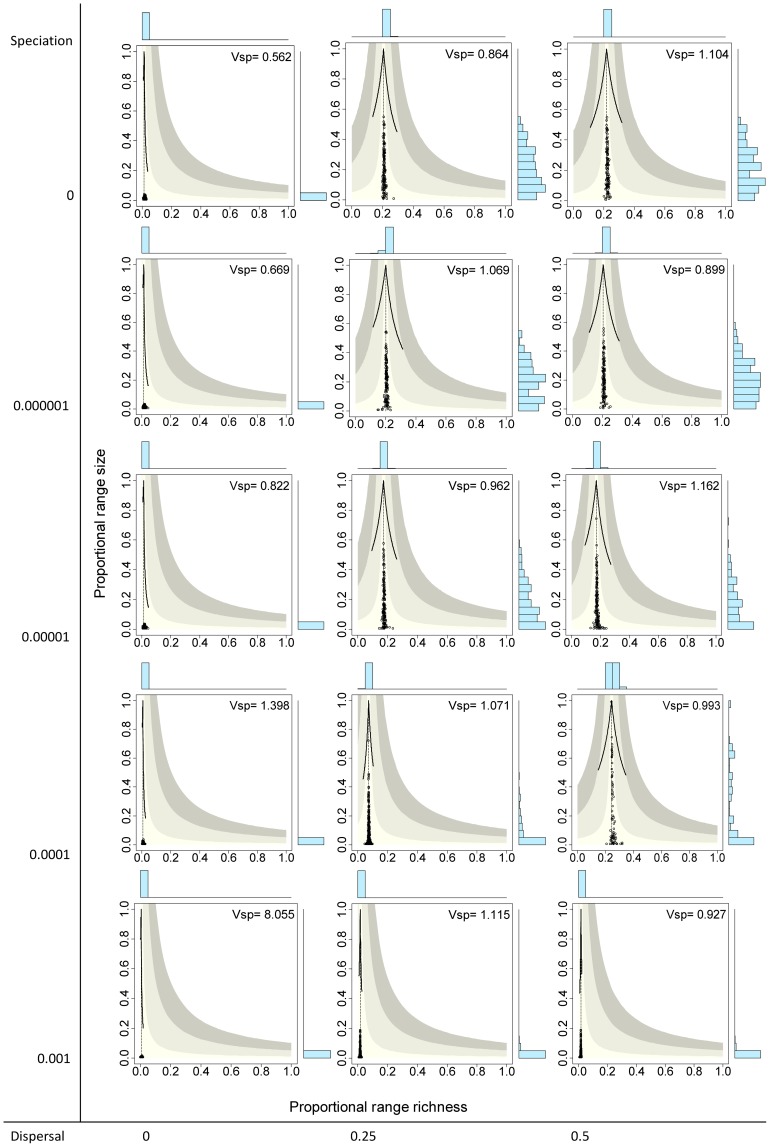
Range-diversity plots by species for different combinations of the speciation and dispersal parameters in neutral models. Information as in Figure 2A.

**Figure 4:**
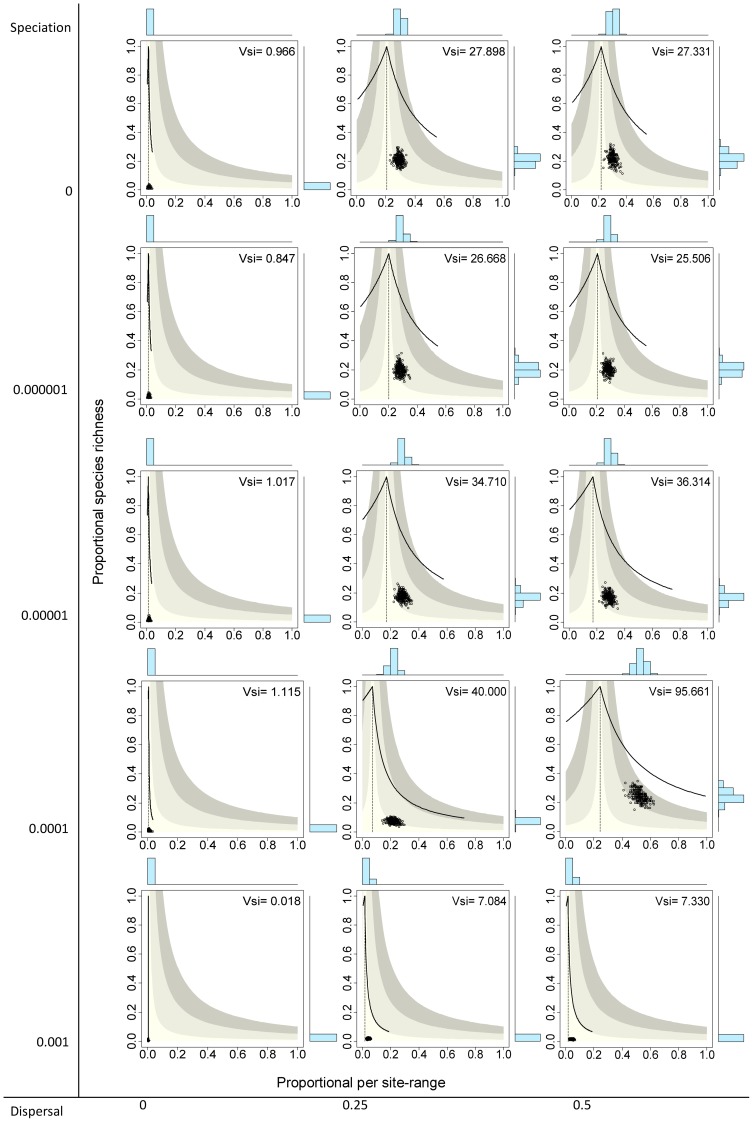
Range-diversity plots by sites for different combinations of the speciation and dispersal parameters in neutral models. Information as in Figure 2B.

**Figure 5:**
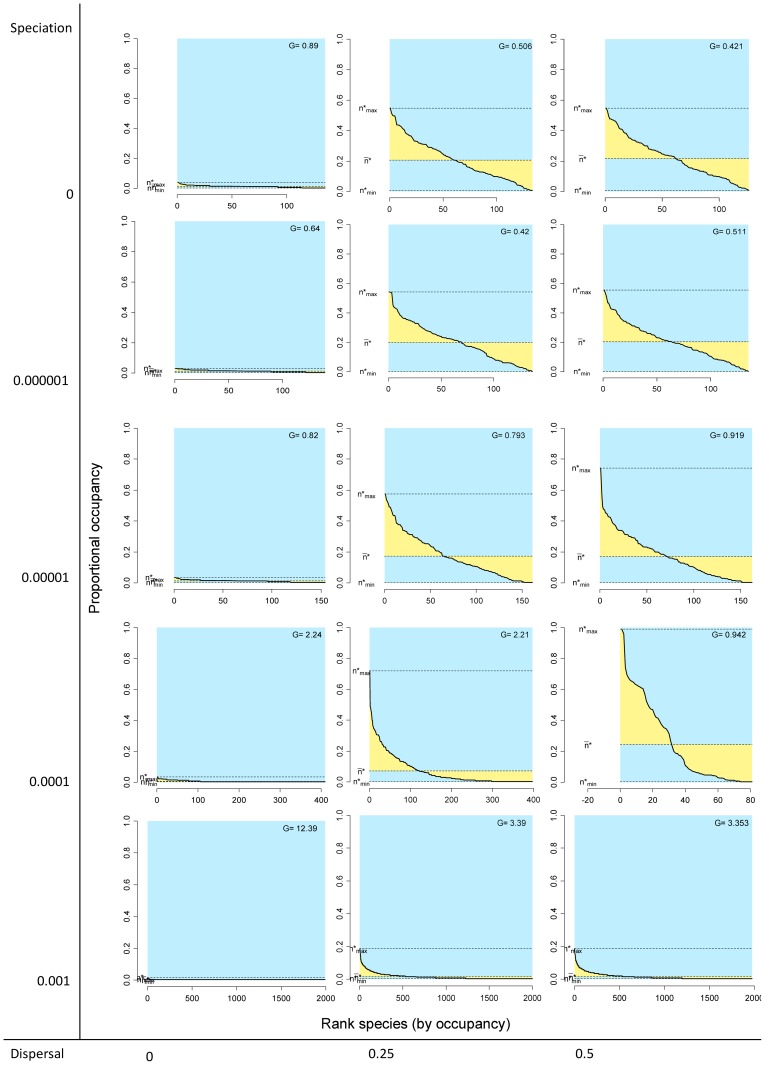
Rank distribution plots by species for different combinations of the speciation and dispersal parameters in neutral models. Information as in Figures 1 and 2C.

**Figure 6:**
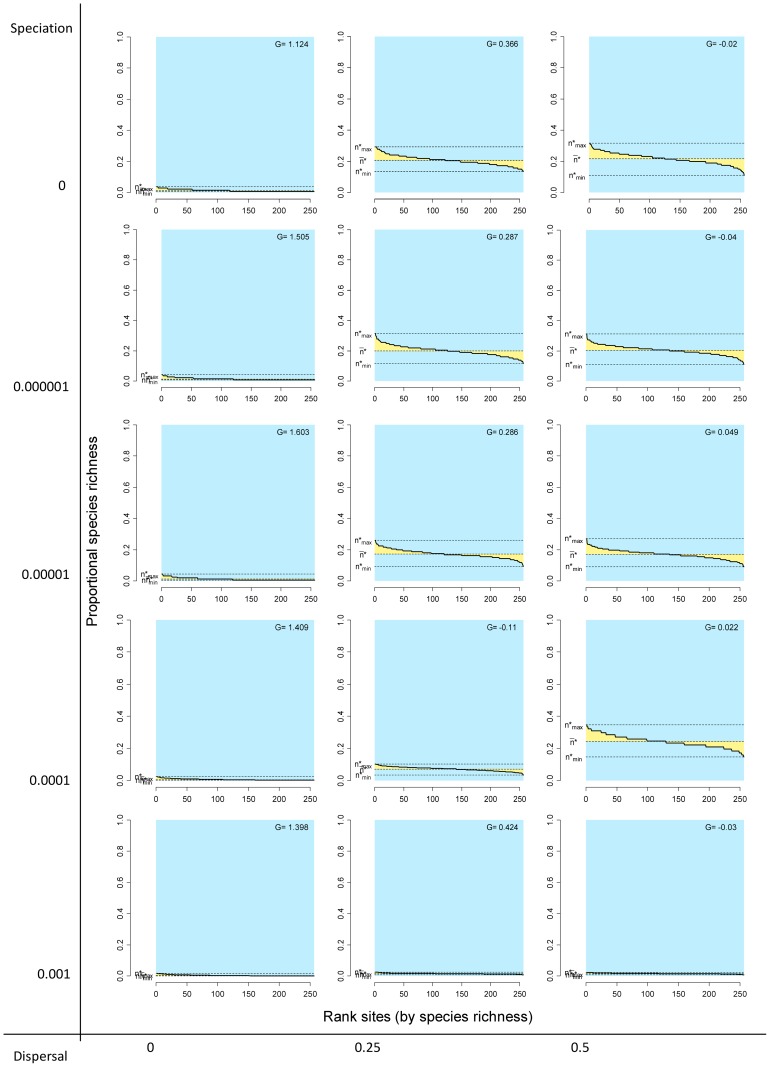
Rank diversity plots by sites for different combinations of the speciation and dispersal parameters in neutral models. Information as in Figures 1 and 2D.

The extreme case with no speciation (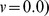 and no dispersal (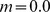) yielded systems with the 136 species having very small ranges, producing presence-absence matrices with very low fill (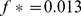), meaning that on average only 1.3% of species occurred in a given cell and 1.3% of sites were included in the range of a given species. Points in the RD plot for species are concentrated on the lower sector of the graph and along the vertical line, indicating that all species had very small ranges and average covariance close to zero (Fig. 3 top left panel). Similarly, points for sites concentrated on the lower left sector of the RD plots, indicating very low species richness values and average covariance with other sites close to zero (Fig. 4 top left panel). The resulting rank plots were extremely flat, indicating the very low degree of variation both in range size of species and in species richness of sites (Figs. 5 and 6, top left panels).

Systems with no dispersal (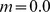) and increasing rates of speciation (from 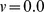 to 0.001) included progressively more species (from 136 to 1992, Table 1), but in all cases the fill of the matrix was extremely low, meaning that all species had very small ranges and all sites had very low species richness, and consequently all points concentrated on the lower left sector of the RD plots, both for species and for sites, regardless of the total number of species (Figs. 3 and 4, left panels). In all these cases, rank plots both for species and for sites were very flat and located on the lower part of the graph (Figs. 5 and 6, left panels).

**Table 1:** Species richness in neutral model simulations with different rates of speciation and dispersal.

	Dispersal rate ( 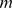 )		
Speciation rate ( 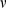 )	0.0	0.25	0.5
0.0	136	133	126
0.000001	139	137	135
0.00001	154	161	162
0.0001	409	397	81
0.001	1992	1999	1874

Simulations with no speciation (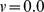) and increasing levels of dispersal (from 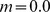 to 0.5) generated systems with increasing fill of the matrix and with higher variation in the range size of species, as evidenced in the histograms of RD plots in the top panels in Fig. 3 and in the rank plots for species in Fig. 5. In the RD plots by species when 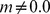, points are arranged along the vertical line, indicating that the average covariance of species is close to zero, a fact also shown by the 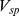 values very close to 1.0 (0.864, 1.104). In these cases, rank plots by species show increased levels of unevenness in range size values but with a nearly symmetric distribution of values. Sites, in contrast, showed less variation of species richness values and overall positive covariance, producing RD plots with clusters of points to the right of the vertical line, and rather flat but symmetric rank plots (Figs. 4 and 6, top panels).

Cases in which both speciation and dispersal differed from zero produced more complex systems. The resultant number of species in the system increased with higher speciation rates, but also was affected by dispersal, with fewer species resulting from higher dispersal values (Table 1). Except for the cases with the highest dispersal levels (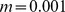), range size varied widely, showing asymmetry in its frequency distribution, particularly for intermediate levels of dispersal (Figs. 3 and 5). Despite the variation in range size, species showed little or no association or segregation, as indicated by points in the RD plots arranging along the vertical zero-covariance line (Fig. 3). Sites showed little variation in their species richness values, producing clusters of points in the RD plots (Fig. 4). These clusters were located in all cases to the right of the vertical zero-covariance line, indicating a general positive covariance of sites in terms of species composition; in one case (when 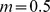 and 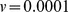), most points located in the sector for average covariance >0.05. Flat rank plots by sites evidenced symmetric species richness frequency distributions showing very little variation (Fig. 6).

### Empirical patterns

**Figure 7:**
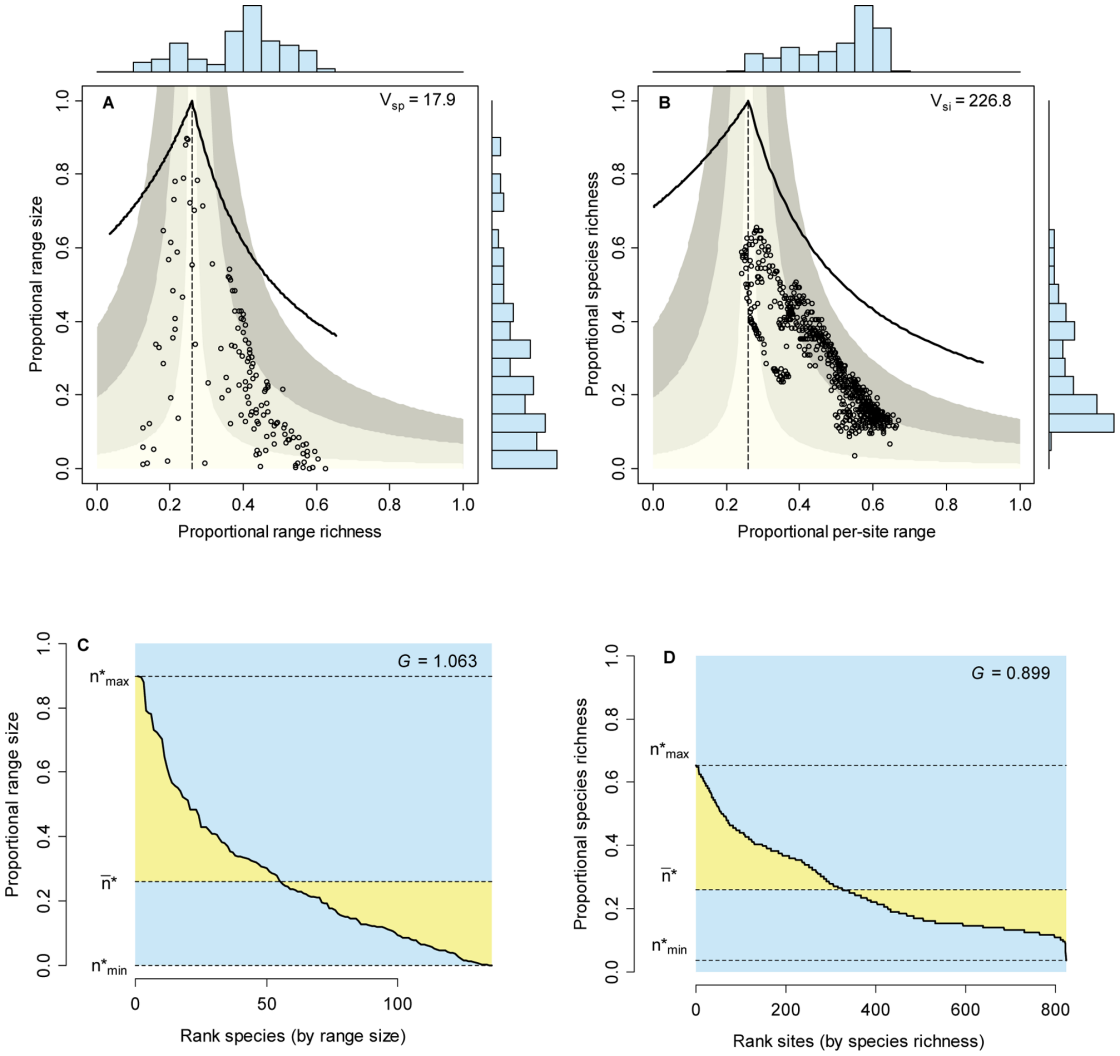
Range-diversity and rank plots for the set of Mexican bats. Information on RD plots (A and B) as in Figure 2A and B; information on rank plots as in Figures 1, 2C, and D.

**Figure 8:**
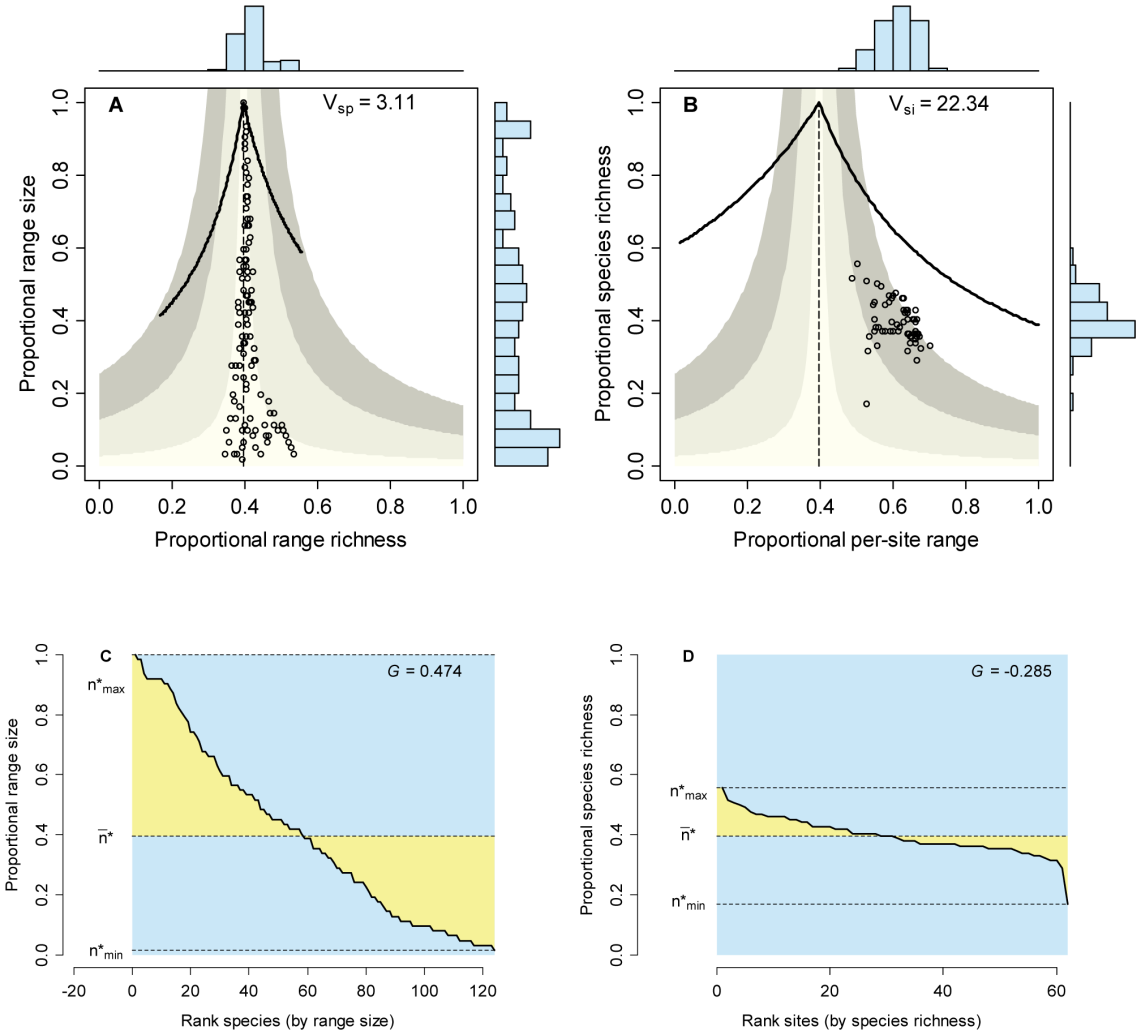
Range-diversity and rank plots for the set of non-volant mammals of central Mexico. Information on RD plots (A and B) as in Figure 2A and B; information on rank plots as in Figures 1, 2C, and D.

Patterns of distribution and diversity for the set of Mexican bats and for the mammals of central Mexico were in appearance similar to those of the neutral model with intermediate levels of dispersal and speciation, with points for species arranging close to the vertical zero-covariance and points for sites forming clusters in the positive covariance sector of RD plots (Figs. 7 and 8). The range size frequency distribution for Mexican bats (Fig. 7A and C), for example, could not be distinguished from the equivalent distribution in the simulation with 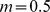 and 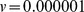 (two-sample Kolmogorov-Smirnov test, two-tailed, *D* = 0.367, *P* = 0.083), and the species richness frequency distribution for this simulation did not differ from that corresponding to the mammals of central Mexico (Fig. 8B and D; *D* = 0.182, *P* = 0.072). A closer look, however, revealed significant differences when comparing the corresponding frequency distributions for bat species richness and for range size of mammals of central Mexico (in both cases, *P*<10^−6^). Moreover, points in RD plots by species scattered farther from the central line in the two empirical cases than in the simulations, generating wider histograms for range richness and higher 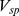 values (Figs. 7A and 8A). Likewise, points for sites in the corresponding RD plots were more scattered in the empirical cases than in the simulations, and extended even to the area of mean covariance >0.1 (Figs. 7B and 8B).

## Discussion

### Initial conditions

Initial conditions give us a glimpse of a system with no ecological or evolutionary processes and totally ruled by chance. Under these extreme conditions, species and sites are expected to be statistically independent among each other in terms of their range size and species richness values, respectively. Thus, this biologically unreasonable model serves not to test the significance of real world patterns but to examine the performance of the analytical tools for a fully randomized case (Fig. 2). Specifically, the model shows that the average covariance among sites and among species is close to zero because sampling units (species and sites) are statistically independent as determined by the randomization. Statistical error in the sampling procedure generated a low level of variation around expected values of range size (20.7% of sites) and species richness (20.7% of species) which produced flat rank plots with very thin yellow areas (Fig. 2 C and D). Average covariance between pairs of species and between pairs of sites was indeed equal to zero, as shown by the points in RD plots arranging along the vertical dashed lines in Fig. 2A and B and by the values of Schluter's 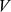 being equal to 1.0. The unrealistic random assignment of individuals to sites and species do not provide an adequate null model for real world patterns, but allows the examination of conditions before the processes defined by the neutral models have any effect on patterns of diversity and distribution. After running the neutral models, any deviation from the patterns generated by the randomized model will indicate the effect of the biological processes simulated in the neutral models.

### The effect of speciation and dispersal

Neutral models allow the incorporation of the stochastic processes of speciation and dispersal that modify the fully randomized initial conditions. Recent research has shown that varying the rules of dispersal in neutral models can produce patterns that differ considerably from the predictions established by early models and that, in some cases, are more similar go real world patterns [12], [34]. However, these models incorporate traits of species, such as dispersal capability, or of sites, such as probabilities of receiving dispersing individuals, that depart from the original concept of neutral models that do not consider any difference among species. To avoid confounding patterns generated by the neutral processes with those generated by differences among species or sites, we chose to limit our analyses to models as originally conceived.

When both speciation and dispersal were set to zero (Figs. 3 to 6, top left panels), the system was driven solely by the effect of the stochastic death-birth process. This process can lead some species to extinction, but cannot generate new species and only changes the distribution of existing species through local extinction or species replacement. Under these circumstances, results of simulations yielded systems with very few species that were very sparsely distributed, so the overall average local species richness and average range size were both very low (1.3% of species and 1.3% of sites, respectively). In the absence of dispersal, the events at each site are independent of changes in other sites, so the average covariance between pairs of sites has to be null. Similarly, because the distribution of each species is independent of what happens with other species, species are expected to represent statistically independent units.

The location of points in range-diversity plots reveals all these patterns (Figs. 3 and 4, top left panels). Points for species are few, concentrated on the lower part of the plot (low species richness), and arranged along the vertical line (zero average covariance, meaning no association or segregation). The number of sites remains the same in all simulations, but in this case the corresponding points are also located in the lower part of the plot and along the vertical line, indicating cells with low species richness and null association between each other. The high concentration of points in the RD plots and the rather flat rank curves (Figs. 5 and 6, top left panels) show the low variation in range size among species and of species richness between sites.

Simulations with 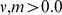 showed the combined effect of speciation and dispersal on patterns of distribution and richness. Speciation alters patterns of diversity by two mechanisms: Besides its direct role in balancing extinction to maintain biological diversity by generating new species [8], speciation also has an effect on the patterns of range size by fostering species with small ranges, thus producing asymmetrical RSFDs, as evidenced by the higher skewness in simulations with higher speciation rates (Figs 5 and 6). In runs of the model with 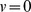, extinction of species was frequent, species richness was low (Table 1), and the RSFD tended to be truncated, having few or none widespread species and showing low skewness (Figs. 4 and 6). Intermediate levels of speciation produced RSFDs with longer tails towards larger ranges.

In dynamical models of diversity, dispersal has the direct effect of increasing the ranges of species and the indirect effect of diminishing differences in composition among sites [35]. We detected this effect in the higher 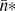 values, and thus of 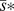 values, at higher levels of dispersal. This trend can be seen in RD plots with the vertical line located more to the right (higher 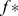 values) in simulations with higher levels of dispersal (Figs. 3 and 4). In extreme cases, very high levels of dispersal can lead to a pattern in which every species occurs in all sites, in which case all species richness values and all range sizes are exactly equal. Such case would generate RD plots with all points overlapped at the extreme top right part of the graph, and rank plots with the curve along the top limit.

The combined effects of speciation and dispersal determine the final outcome in terms of species richness and the shape of the RSFD. In general, higher rates of speciation and lower rates of dispersal generated systems with higher species richness and RSFDs that were more positively skewed (that is, with a higher number of restricted ranges), but the combined effect is more evident at intermediate values of speciation and dispersal. Because of the mathematical relationship between parameters of diversity and distribution, a similar combined effect of speciation and dispersal can be seen in the SRFDs (Figs. 4 and 6). Again, higher speciation and lower dispersal tend to generate SRFDs that are more skewed, but the effect is less noticeable than for the RSFDs.

### Patterns of co-occurrence and co-diversity

Our simulations generated distributional ranges that were independent of each other, as indicated by the 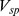 values being close to 1.0 and by the mean of the variance-covariance matrix in all cases being <0.001. As shown by the RD plots, stochastic deviations from this pattern depend to a large extent on the number of species and on the shape of the range-size and species-richness frequency distributions (Fig. 3). In all cases, points arranged along the vertical line determined by 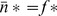, indicating that the average covariance of single species with the rest of the community was in all cases close to 0.0. The variance in the distribution of each species is determined by its range size, and for presence-absence data it is in fact equal to the binomial variance 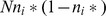, so it is >0 except for species occupying all sites, and is maximal for intermediate range sizes. If variances are positive and the mean of the variance-covariance matrix is close to zero, then at least some of the covariance values have to be negative. These deviations can be seen in the points in RD plots not lying exactly on the vertical line of zero covariance, but remaining very close to it.

Another implication of the relationship between range size and variance is that even if the ranges of species are independent of each other, non-neutral patterns of co-diversity can be generated, in some cases being undistinguishable from empirical patterns [23], [36]. Differences between the results of the neutral models and the initial conditions point to the effect of stochastic processes (as opposite to simple randomizations) in generating non-random patterns even in interaction-free species assemblages. The relationship between variance and covariance by species and by sites can be seen clearly in the patterns of co-diversity in RD plots (Fig. 4). Because dispersal in our models was a function of distance, sites were not statistically independent of each other because closer cells were more likely to interchange species than more distant cells. In simulations with intermediate or high dispersal rates and intermediate speciation, sites clearly deviated to the right from the vertical line indicating zero covariance (Fig. 4) and the system showed very high 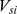 values and low dispersion in the species richness frequency distribution, producing very flat rank curves (Fig. 6).

Simulations with high dispersal rates and intermediate speciation rates show most clearly the interaction between patterns of variance-covariance between species and sites. With 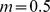 and 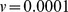, the model generated a RSFD quite similar to the highly skewed curves that characterize natural assemblages, which have many species with small ranges and a few species with very large ranges [31], [32]. The high variance of this kind of distribution is reflected in the variance-covariance matrix by sites, because the sum of elements of this matrix equals the variance in range size of species [33], [37]. The effect on sites is a very high average co-variance, that is, a positive co-diversity that can be seen in the points in the RD plot being shifted to the right of the vertical line and in the area of average covariance between 0.5 and 0.1 (Fig. 4). In the opposite direction, the low variance shown in the SRFD determines a low level of variance-covariance in the ranges of species, resulting in points being arranged along the vertical line marking an average zero covariance (Fig. 3).

Comparison of patterns of co-occurrence and co-diversity in our simulations reveals an asymmetry in the output of neutral models. Neutral models generate systems in which average covariance among species is almost null, but average covariance among sites is always positive. That is, species are independent of each other in their geographic distribution but sites show varying levels of positive similarity.

One could imagine neutral models based on sites instead of on species. In these models, events taking place in each site would be independent of other sites, and the assignment of individuals to species would depend on their phylogenetic closeness. In this case, covariance among sites would be close to zero and covariance among species would be positive. From a mathematical standpoint, these new kind of neutral models would be identical to regular neutral models, because species-based models could generate the results of site-based models simply by transposing the presence-absence matrix. In both kinds of models, the statistical relationships between richness and range size allow the generation of non-random patterns (positive covariances) through the action of stochastic processes involving independent entities (species or sites).

### Comparing neutral and real world patterns

Empirical examples in Figs. 7 and 8 show that neutral models can generate patterns that closely resemble those of real world assemblages. Certain combinations of dispersal and speciation (*e.g*., 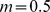, 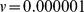) can recreate the range-size frequency distribution for Mexican bats (Fig. 7A, right panel) and the species-richness frequency distribution for mammals of central Mexico (Fig. 8B, right panel). However, the SRFD for bats and the RSFD for mammals differ significantly from the predictions of the neutral model. These two empirical examples show the importance of considering different patterns when comparing neutral models with real life assemblages.

The importance of a multi-pattern approach is even more clear when considering patterns of covariance. The mammals of central Mexico show a very low level of average covariance (co-occurrence), as shown by the points in Fig. 8A arranging along the vertical zero-covariance line and by the relatively low 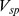 value. However, the corresponding RD plot by sites (Fig. 8B) shows higher values of covariance than those generated by the neutral models. In the case of Mexican bats (Fig. 7), both patterns of covariance (by species and by sites) show much more dispersion than the neutral models. These examples demonstrate the importance of examining patterns of covariance among species and among sites in assessing the performance of neutral models in comparisons with real world assemblages.

All neutral models examined here generate systems in which the species are independent of each other in terms of their distribution, thus producing RD plots with points arranged along the vertical zero-covariance line (Fig. 3). In contrast, models with intermediate and high dispersal values generate systems in which the covariance among sites is positive (Fig. 4). In most cases, average covariances range from 0.01 to 0.05, and in one case covariances range from 0.05 to 0.1 (Fig. 4). These contrasting patterns for species and for sites stress the necessity of incorporating both patterns in comparisons of neutral models with real world assemblages. Examining one or the other in isolation could lead to equivocal results if the chosen pattern happens to resemble the predictions of neutral models.

The variance-covariance matrices for species and for sites are tied by mathematical relationships that determine the overall mean covariance, but the individual pair-wise values of covariance can show a great deal of variation depending on the way species distribute among sites [25], [26]. Thus, deviations of patterns of covariance by sites and by species from expectations of neutral models are evidence of biological factors that modify the patterns of association/segregation among species and of similarity/divergence among sites. In the case of Mexican bats, for example, the positive and negative pair-wise covariances are generated by species distributing mostly in the neotropical region, mostly in the nearctic region, or in the transition zone between the two regions. This kind of patterns, produced by historical or ecological factors, cannot be reproduced with simple neutral models. The examination of RD plots for patterns of co-occurrence of species and co-diversity of sites is therefore a powerful tool in testing the predictions of neutral models.
